# Small bowel involvement is a prognostic factor in colorectal carcinomatosis treated with complete cytoreductive surgery plus hyperthermic intraperitoneal chemotherapy

**DOI:** 10.1186/1477-7819-10-56

**Published:** 2012-04-11

**Authors:** Emmanuel I Benizri, Jean-Louis Bernard, Amine Rahili, Daniel Benchimol, Jean-Marc Bereder

**Affiliations:** 1 Department of General Surgery and Digestive Cancerology, Centre Hospitalier Universitaire de Nice, Hôpital Archet 2, 151 Route de Saint Antoine de Ginestière, Nice Cedex 3 B.P. 3079, France

**Keywords:** Colorectal cancer, Peritoneal carcinomatosis, Prognostic factors, Cytoreductive surgery, Intraperitoneal chemotherapy

## Abstract

**Background:**

Cytoreductive surgery and hyperthermic intraperitoneal chemotherapy (HIPEC) is a promising treatment for patients with peritoneal carcinomatosis (PC). Our objective was to identify new prognostic factors in patients with PC from colorectal cancer treated with this procedure.

**Methods:**

All patients with PC from colorectal cancer treated by HIPEC from January 2000 to December 2007 were prospectively included. The tumor extension was assessed by the Peritoneal Cancer Index (PCI) and the residual disease was recorded using the completeness cytoreductive score (CCs). All clinical and treatment data were computed in univariate and multivariable analyses using survival as primary end point.

**Results:**

We carried out 51 complete procedures in 49 consecutive patients. The mean PCI was 10. The allocation of CCs was: CC-0 = 37, CC-1 = 14. The five-year overall and progression-free survival rate were 40% and 20%, respectively. Several prognostic factors for survival were identified by univariate analysis: PCI < 9 (*P *< 0.001), CC-0 vs. CC-1 (*P *< 0.01) and involvement of area 4 (*P *= 0.06), area 5 (*P *= 0.031), area 7 (*P *= 0.014), area 8 (*P *= 0.022), area 10 (*P *< 0.0001), and area 11 (*P *= 0.02). Only the involvement of the distal jejunum (area 10) was significant in the multivariable analysis (*P *= 0.027).

**Conclusions:**

We demonstrated that the involvement of area 10 (distal jejunum of the PCI score) was an independent factor associated with poor prognosis.

## Background

Peritoneal carcinomatosis (PC) is a common evolution of digestive cancers which affects 10% of patients with colorectal adenocarcinomas at the initial time of diagnosis and 25% of patients with recurrent disease [1]. Moreover, as reported in the French EVOCAPE 1 Study, PC is traditionally associated with a poor prognosis: for colorectal cancer patients, mean and median overall survival were 6.9 and 5.2 months, respectively [2].

For two decades, the development of a new concept involving cytoreductive surgery and hyperthermic intraperitoneal chemotherapy (HIPEC) has demonstrated promising results. One of the first publications, Sugarbaker *et al. *[3] reported a three-year survival rate of 61%. Later, other phase II studies became available showing a median survival lasting from 13 to 63 months [4-8]. A single randomized trial comparing cytoreduction followed by HIPEC and adjuvant systemic chemotherapy to systemic chemotherapy demonstrated the superiority of the combined treatment [9]. All these results suggest that this combined surgical treatment should be considered as the current standard treatment for PC from colorectal origin.

Despite several differences in study design in all publications including variation of chemotherapy regimen, techniques of hyperthermia or duration of the procedure, two prognostic factor are currently identified [10]: the extent of disease before surgery, usually scored by the peritoneal cancer index (PCI), and the quality of the surgical cytoreduction, measured by the completness of cytoreduction score (CCs) [11]. So, only patients with limited PC and complete cytoreduction will really benefit from cytoreductive surgery and HIPEC.

The aim of our study was to identify new prognostic factors in patients treated by cytoredutive surgery and HIPEC for PC from colorectal origin at Nice Peritoneal Cancer Center. Thus, above all, we assessed the prognostic impact of PC involvement by area.

## Methods

From January 2000 to December 2007, all patients with resectable PC from colorectal cancer underwent cytoreductive surgery followed by HIPEC at Nice Peritoneal Cancer Center. All patients were included in a prospective database, and gave their signed informed consent. This study was approved by our institutional review committee.

### Inclusion criteria

All primary cancers were confirmed by a biopsy.

In order to evaluate the extent of the disease, all patients underwent preoperative investigations which included thoracic, abdominal and pelvic computed tomography (CT) with oral and intravenous contrast agents. From 2004, positron emission tomography was additionally performed.

Anaesthetic evaluation, echocardiography, and spirometry were performed for all patients.

Patients were then selected preoperatively according to the following criteria: (1) < 75 years old and good general status (World Health Organization (WHO) Performance Status < 2); (2) PC from colorectal carcinoma, but non-appendiceal; (3) no extra-abdominal disease; (4) no multiple, diffuse, and huge-tumor peritoneal deposits on the CT scan; (5) no evidence of intestinal obstruction or involvement; (6) no evidence of biliary or ureteral obstruction; and (7) no massive and total abdominal involvement on clinical examination.

For borderline cases, a laparoscopy was performed preoperatively.

### Modalities of the combined treatment

**Figure 1:**
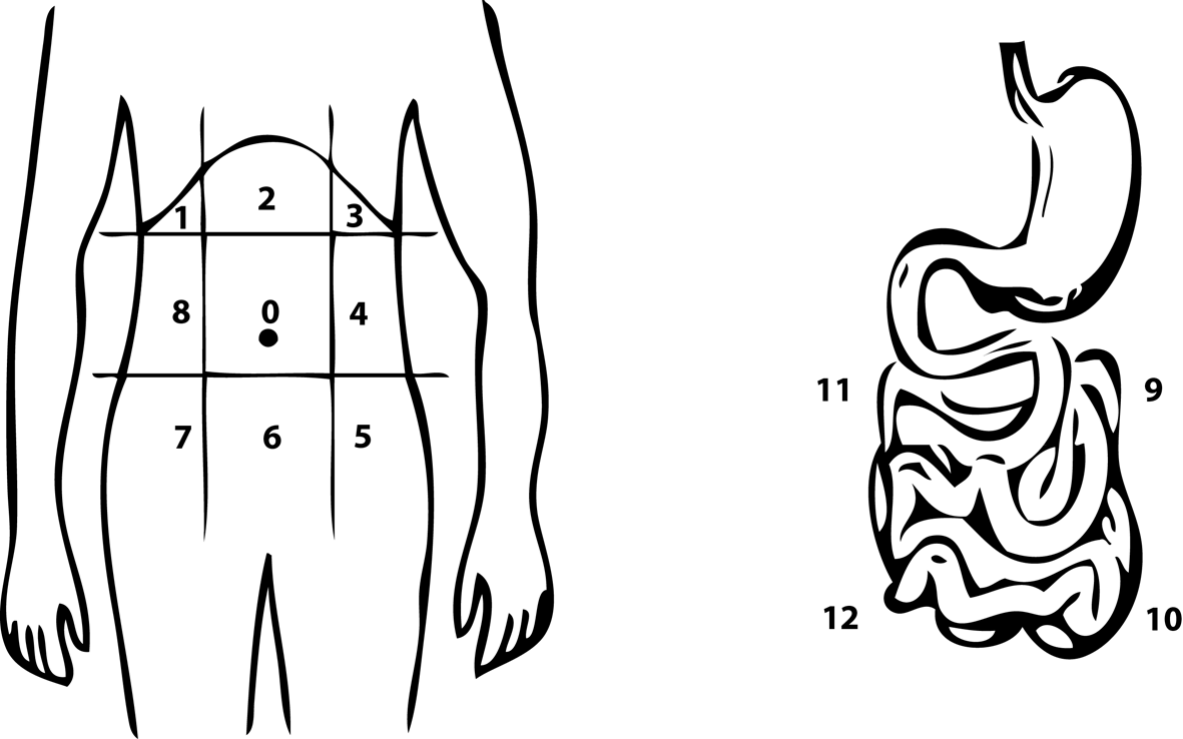
**Description of the PCI (Peritoneal Cancer Index)**. The PCI provides an assessment of tumor volume and distribution in the abdominopelvic cavity (nine quadrants) and the small bowel (four regions).

During the laparotomy, the extent of the PC was calculated for each patient using the PCI, as described by Sugarbaker *et al. *[11]. This score links the tumor location (areas 0 to 8 for the abdominal cavity and 9 to 12 for the small bowel) with the lesion size (LS0: no tumor deposit, to LS3: tumor thickness > 5 cm). So, the PCI can range from 1 to 39 (Figure 1). If the PC seemed to be resectable, a cytoreductive surgery was done with resection or destruction by electrovaporation of all the macroscopically detectable peritoneal disease. After this surgical procedure, the CCs was evaluated for each patient. CC-0 score indicated that no tumor was visible in the peritoneal cavity; CC-1 indicated a residual tumor < 2.5 mm; CC-2 indicated a residual tumor between 2.5 mm and 2.5 cm; CC-3 indicated a residual tumor > 2.5 cm or a confluence of nodules [11].

Only patients scoring CC-0 or CC-1, were considered to have a complete cytoreduction and were eligible for the HIPEC procedure. Mitomycin C was administrated intraperitoneally in the open abdominal cavity using the coliseum technique with a Thompson self-retaining retractor [12]. The dose was 12.5 mg/m^2 ^for men and 10 mg/m^2 ^for women, in 2 L/m^2 ^of 1.5% dextrose peritoneal dialysis solution. A heat exchange kept the intraperitoneal temperature at 42°C for 90 min.

### Follow-up and study methods

All patients were followed every 6 months with a clinical examination, thoracic, and abdominopelvic CT scan, and carcinoembryonic antigen measurement. All data were collected in a prospective database.

The statistical analysis used overall survival as the primary endpoint.

For categorical variables, the χ^2 ^test or Fisher's exact test, when appropriate, was used. Continuous variables were compared with the student *t*-test.

Survival was defined from the time of the surgical procedure. Survival analysis was performed using the Kaplan-Meier method, and compared using the log-rank test. No patient was excluded from survival analysis.

A multivariate analysis using a Cox-regression model was done to identify independent prognostic factors for survival.

*P *values of less than 0.05 were considered significant.

Statistical analysis was performed using SPSS v 16 for Windows (SPSS, Chicago, Illinois, USA).

## Results

### Descriptive data

From January 2000 to December 2007, 51 procedures (HIPEC) in 49 consecutive patients were performed. There were 30 women (61.2%) and 19 men (38.8%). The mean age was 52.7 ± 11 years (range: 32-75). The location of the tumor was colonic in 41 cases (83.7%) and rectal in all the others cases. At initial staging, 22 patients (44.9%) had positive lymph nodes; tumor grade was poorly differentiated for six patients (12.2%), moderately differentiated for 35 patients (71.4%) and well differentiated for eight patients (16.3%). The time between the initial diagnosis of colorectal cancer and HIPEC procedure was 20 ± 9 months (range: 1-87). Thirty-seven (72.5%) patients received neoadjuvant chemotherapy (with a mean of 1.2 lines (range: 0-4)). The mean PCI was 10 ± 6.2 (range: 1-22). The number of areas involved was 5 ± 3.3 (range: 1-13). Thirty-seven procedures (72.5%) were considered as CC-0 cytoreduction; 12 procedures (23.5%) were CC-1 cytoreduction. The CCs was significantly correlated with the PCI: median PCI was 7 in the CC-0 group versus 16 in the CC-1 group (*P *< 0.0001). During the cytoreductive surgery, there were 3 ± 1.6 visceral resections (range: 0-7). Among the 31 patients (61.8%) with small bowel involvement, 19 (61.3%) had a small bowel resection and 12 (23.5%) underwent destruction of the peritoneal nodules by electrovaporation. The operative time was 474 ± 127 min (range: 180-780). The mean unit of blood transfusion was 1.3 ± 1.6 (range: 0-7).

There was no mortality. Ten patients (20.5%) had a complication which required specific medical or surgical treatment (grade III and IV of Clavien). These adverse events included digestive fistula (*n *= 2), biliary leak (*n *= 2), bleeding (*n *= 1), short bowel with diarrhea (*n *= 2), subphrenic abcess (*n *= 1), and thrombocytopenia (*n *= 1). The length of hospital stay was 18.6 ± 10 days (range: 7-52).

### Survival data

**Figure 2:**
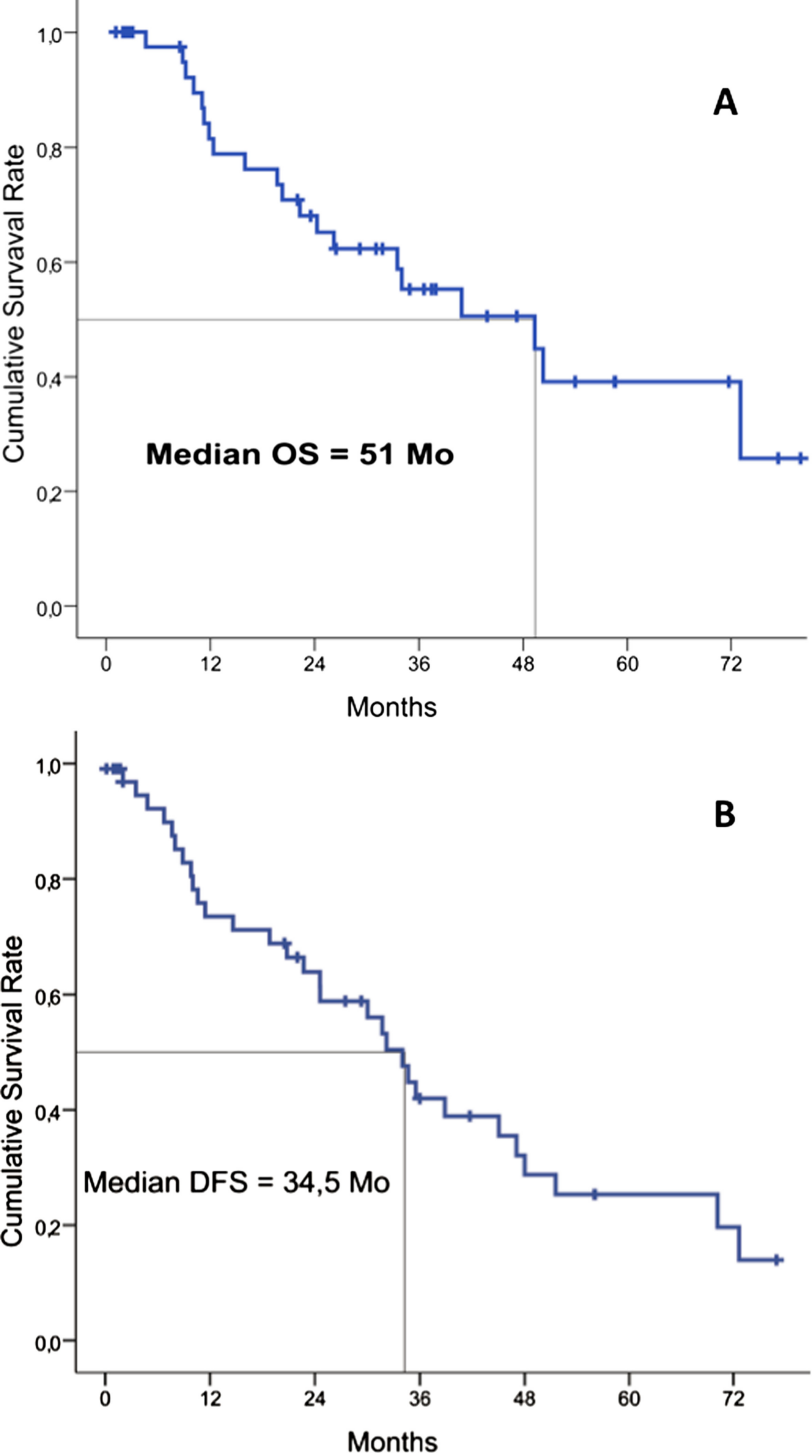
**Survival results in 49 patients after HIPEC (hyperthermic intraperitoneal chemotherapy) for colorectal peritoneal carcinomatosis**. Overall survival (OS). Disease free survival (DFS).

The mean follow-up was 27 ± 8 months (range 9-83). The median overall survival was 51 months and the median disease-free survival was 34.5 months (Figure 2A and 2B).

**Figure 3:**
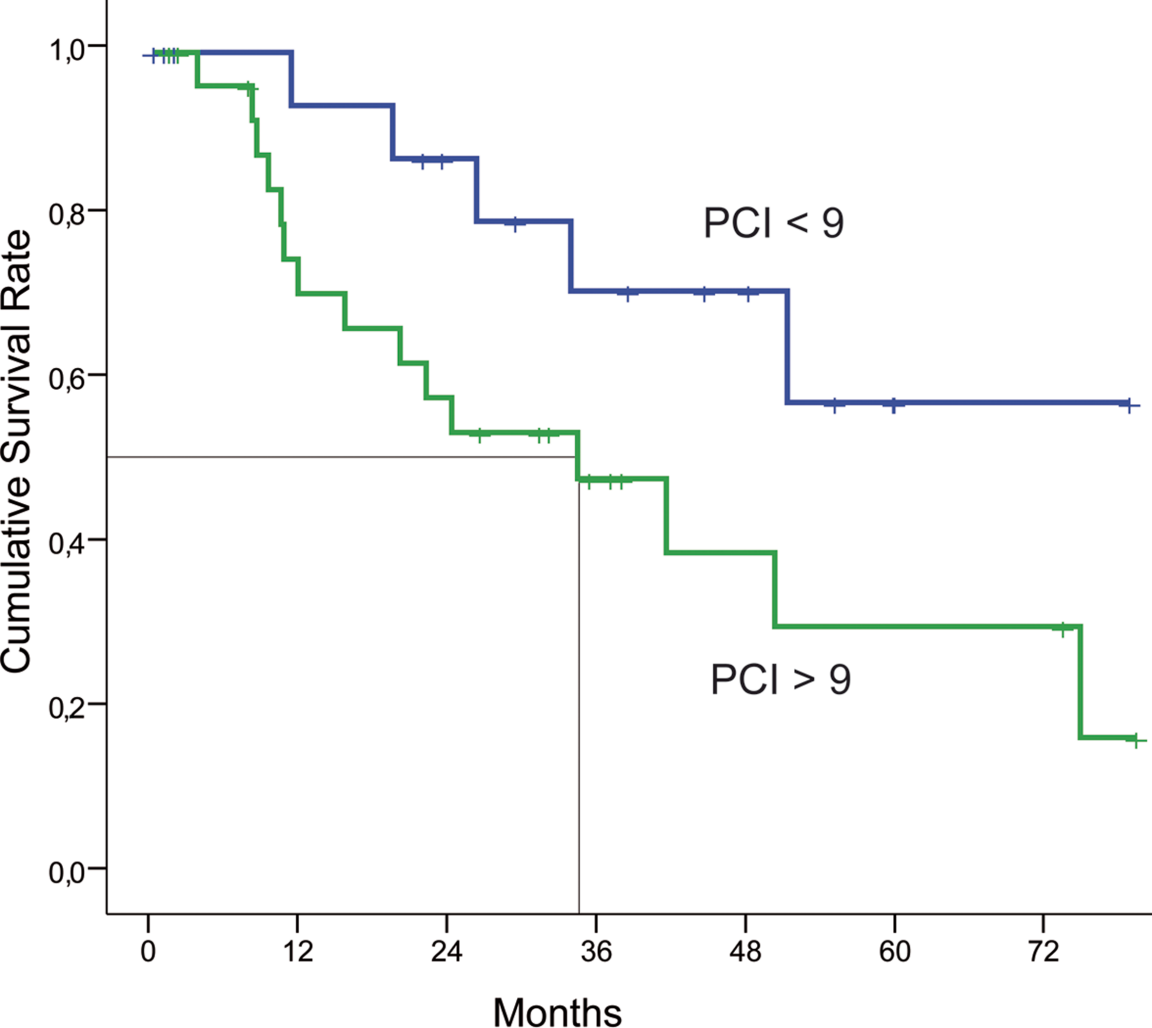
**Kaplan-Meir survival curves for patients with PCI (Peritoneal Cancer Index) > 9 or < 9**. In univariate analysis, the PCI is a prognostic factor (Log rank: *P *< 0.001).

**Figure 4:**
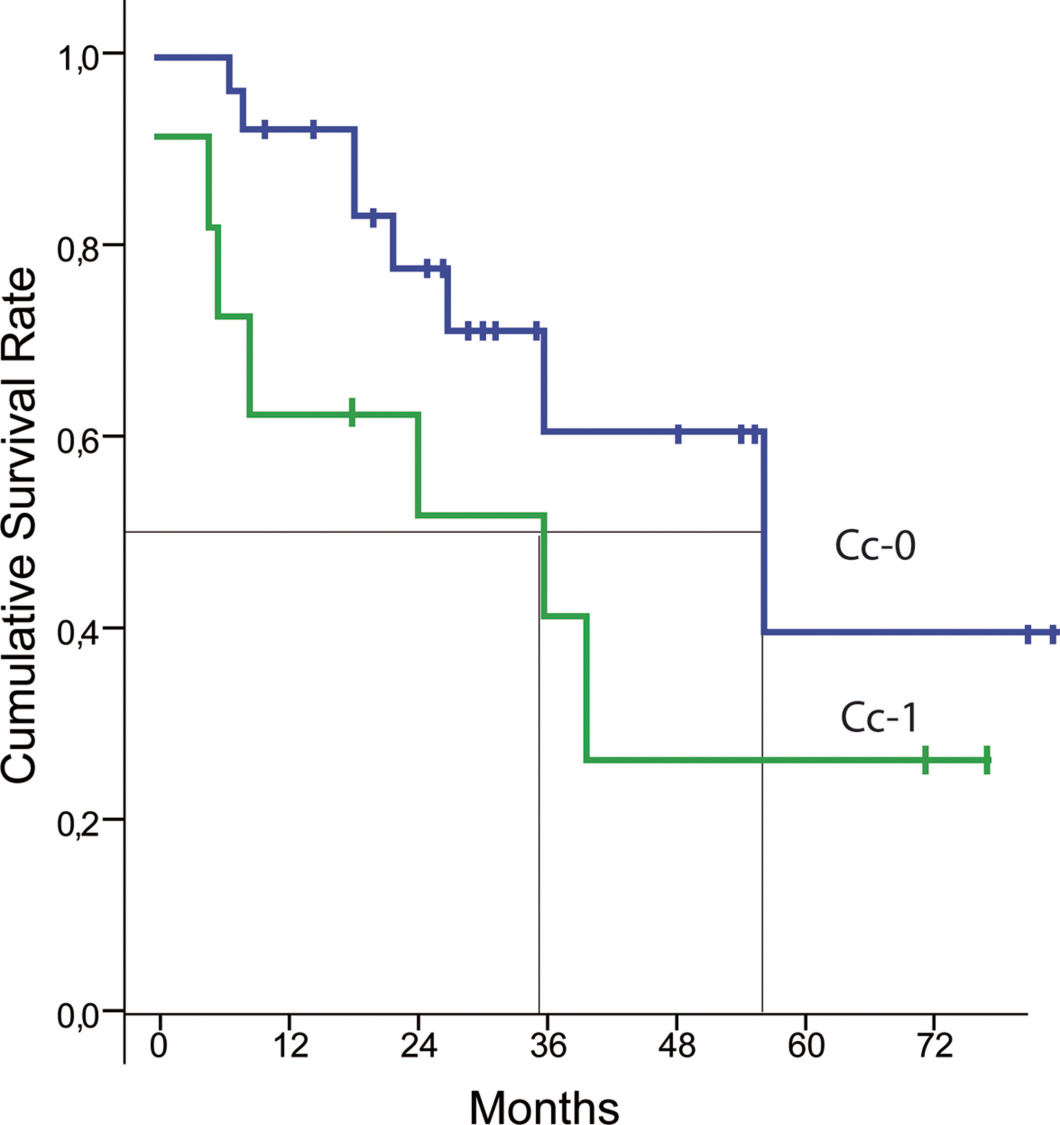
**Kaplan-Meir survival curves for patients Cc0 or Cc1 (Cytoreductive score 0 or 1)**. In univariate analysis, the CCs is a prognostic factor (Log rank: *P *< 0.01).

In univariate analysis, the extent of the peritoneal carcinomatosis mesured by the PCI (*P *< 0.01) and the quality of the cytoreduction measured by the CCs (*P *< 0.01) were significant prognostic factors (Figures 3 and 4). Moreover, the involvement of the areas 4, 5, 7, 8, 10, and 11 was also associated with **median **survival in univariate analysis (Table 1). Age (*P *= 0.87), sex (*P *= 0.48), preoperative chemotherapy (*P *= 0.29), grade of the primary tumor (*P *= 0.13), and nodal status (*P *= 0.85) were not associated with median survival in univariate analysis.

**Table 1:** Univariate analysis of survival according to the areas involved in peritoneal carcinomatosis

Variable: area involved	Number of patients (%)	*P *value
0 (Umbilical region)	26 (51)	0.051

1 (Right upper quandrant)	20 (39.2)	0.931

2 (Epigastrium)	16 (31.4)	0.191

3 (Left upper quandrant)	11 (21.6)	0.667

4 (Left flanc)	26 (51)	0.006

5 (Left iliac fossa)	26 (51)	0.031

6 (Hypogastric region)	37 (72.5)	0.189

7 (Right iliac fossa)	29 (56.9)	0.014

8 (Right flanc)	20 (39.2)	0.022

9 (Proximal jejunum)	7 (13.7)	0.882

10 (Distal jejunum)	17 (33.3)	< 0.0001

11 (Proximal ileum)	16 (31.4)	0.020

12 (Distal ileum)	13 (25.5)	0.297

In multivariate analysis by Cox model represented in Table 2, the involvement of area 10 was the only significant prognostic factor (HR = 21.81 CI 95 (1.42-334.5) *P *= 0.027).

**Table 2:** Identification of independent prognostic factors for survival by multivariate analysis using a Cox regression model

Risk factors	Hazard Ratio (95% CI)	*P *value
Peritoneal Cancer Index (PCI)	1.76 (0.26-2.19)	0.32

Completeness of Cytoreduction Score (CCs)	0.43 (0.05-3.39)	0.42

Area involved		

Area 4	1.79 (0.43-7.54)	0.43

Area 5	3.64 (0.68-19.46)	0.13

Area 7	2.07 (0.51-8.34)	0.31

Area 8	3.24 (0.49-21.47)	0.22

Area 10	21.81 (1.42-334.5)	0.027

## Discussion

For many years, PC was considered an incurable disease. The development of cytoreductive surgery followed by HIPEC has totally changed the course of this condition, with median survival reported lasting from 13 to 63 months in selected cases [4-8]. In this study, the overall median survival was 51 months, which supports the effectiveness of this technique. Currently, the objective is to optimize the selection of patients who will benefit from this aggressive approach. In this context, many prognostic factors have been described to predict the outcome of HIPEC procedures.

The CCs is usually reported as the most important factor in predicting outcome. Survival results following CC-2 cytoreduction are very poor, and HIPEC should not be performed in this situation [13,14]. In the study reported by Glehen [5], the five-year survival rate was 31% for CC-0 patients. These results were significantly better than CC-1 (15%) or CC-2 (no patients alive) conditions. In univariate analysis, our results confirm the data in the literature with a median survival of 52 months for CC-0 vs 35 months for CC-1 patients (*P *< 0.01). Nevertheless, this prognostic factor was not demonstrable in our multivariate analysis probably because we do not perform HIPEC for CC-2 patients.

In addition, the extent of the peritoneal carcinomatosis measured by the PCI is reported as a prognostic factor [15]. One problem with the PCI is to define the cutoff associated with a poor prognosis. This limit ranges between 10 and 20 depending on the authors [15-17]. Our study confirmed in univariate analysis that the extent of disease before surgery is a prognostic factor with a threshold of 9, under which patients have an excellent prognosis. Moreover, as described in Figure 2, the CCs was significantly correlated with the PCI, justifying that these two factors are not highlighted in multivariate analysis. Finally, PCI is a score calculated from qualitative and quantitative data. However, in published series, this score is considered as purely quantitative data which induce interpretation bias. Therefore, we felt it was important to assess only the impact of qualitative data, by studying the location of PC. Our results were very interesting since we have shown for the first time that the involvement of the small bowel, and especially in area 10, was an independent prognostic factor of survival.

The involvement of the small bowel is a well-known cause of incomplete surgical cytoreduction, especially when the disease is located at the junction between the small bowel and its own mesenterium [18]. Nevertheless, even when the surgical cytoreduction is complete, this spread appears to be an independent prognostic factor. The invasion of the rest of the small bowel (areas 9, 11, and 12) did not emerge in this study, and may be due to lack of power.

However, it is very difficult to appreciate preoperatively the precise extension of the CP, especially for the small bowel. CT scan is the most used imaging method, but in fact, it is inaccurate and its impact on the management of patients is modest [19]. Laparoscopy may supplement imaging modalities, but in our practice, it is sometimes difficult to perform (iterative surgery, adhesions) and it is not always accurate (assessment of the retroperitoneal space and the posterior segments of the liver). However, this tool should not be disregarded, and its use can be discussed as part of a multidisciplinary approach if diffuse or massive small bowel involvement is suspected preoperatively [20].

Lymphatic drainage of the peritoneal cavity is ensured through the so-called stomata which facilitate communication between the abdominal cavity, and the submesothelial diaphragmatic lymphatics [21]. The stomata of the visceral and parietal peritoneum are very heterogeneous, suggesting different functional implications. The presence of openings in the mesentery explains an increased lymphatic permeability [22]. This could lead to a lymph node extension, and explain the poor prognosis associated with the invasion of the small bowel.

In this context, lymph node involvement is also a prognostic factor reported in some series [23]. This issue has not been found in our study. The lymphatic extension reflects systemic spread of disease that cannot be treated with a locoregional therapeutic approach. Thus, the involvement of the small bowel, even in the absence of lymph node metastases, could suggest additional treatment with systemic chemotherapy.

Other, predictors are reported in the literature such as the differentiation grade or the location of the primary tumor [16]. These factors appear less strong, and were not found in our series.

## Conclusion

We have identified several prognostic factors by univariate analysis, especially the PCI, the CCs and some areas of the peritoneal cavity. But most interesting is the fact that only the invasion of the distal jejunum (area 10) is independently associated with a poor prognosis. It will be difficult to assess this invasion preoperatively because conventional imaging techniques underestimate the extent of intraperitoneal spread. Nevertheless, in case of small bowel invasion confirmed at surgery, additional treatment with systemic chemotherapy could be proposed.

## Abbreviations

CCs: Completeness cytoreductive score; DFS: Disease free survival; HIPEC: Hyperthermic intraperitoneal chemotherapy; OS: Overall survival; PC: Peritoneal carcinomatosis; PCI: Peritoneal Cancer Index

## Competing interests

The authors declare that they have no competing interests.

## Authors' contributions

EIB: study conception and design, acquisition of data, analysis and interpretation of data, drafting of manuscript. JLB: study conception and design, critical revision. AR: acquisition of data, analysis and interpretation of data, critical revision. DB: study conception and design, critical revision. JMB: analysis and interpretation of data, critical revision. The final version of this paper has been seen and approved by all authors.
